# A Comprehensive Reference Transcriptome Resource for the Common House Spider *Parasteatoda tepidariorum*

**DOI:** 10.1371/journal.pone.0104885

**Published:** 2014-08-13

**Authors:** Nico Posnien, Victor Zeng, Evelyn E. Schwager, Matthias Pechmann, Maarten Hilbrant, Joseph D. Keefe, Wim G. M. Damen, Nikola-Michael Prpic, Alistair P. McGregor, Cassandra G. Extavour

**Affiliations:** 1 Johann-Friedrich-Blumenbach-Institute for Zoology and Anthropology, Department of Developmental Biology, Georg-August-University Göttingen, GZMB Ernst-Caspari-Haus, Göttingen, Germany; 2 Department of Organismic and Evolutionary Biology, Harvard University, Cambridge, Massachusetts, United States of America; 3 Cologne Biocenter, Institute of Developmental Biology, University of Cologne, Cologne, Germany; 4 Department of Biological and Medical Sciences, Oxford Brookes University, Oxford, United Kingdom; 5 Department of Genetics, Friedrich Schiller University Jena, Jena, Germany; Georg August University of Göttingen, Germany

## Abstract

*Parasteatoda tepidariorum* is an increasingly popular model for the study of spider development and the evolution of development more broadly. However, fully understanding the regulation and evolution of *P. tepidariorum* development in comparison to other animals requires a genomic perspective. Although research on *P. tepidariorum* has provided major new insights, gene analysis to date has been limited to candidate gene approaches. Furthermore, the few available EST collections are based on embryonic transcripts, which have not been systematically annotated and are unlikely to contain transcripts specific to post-embryonic stages of development. We therefore generated cDNA from pooled embryos representing all described embryonic stages, as well as post-embryonic stages including nymphs, larvae and adults, and using Illumina HiSeq technology obtained a total of 625,076,514 100-bp paired end reads. We combined these data with 24,360 ESTs available in GenBank, and 1,040,006 reads newly generated from 454 pyrosequencing of a mixed-stage embryo cDNA library. The combined sequence data were assembled using a custom *de novo* assembly strategy designed to optimize assembly product length, number of predicted transcripts, and proportion of raw reads incorporated into the assembly. The *de novo* assembly generated 446,427 contigs with an N50 of 1,875 bp. These sequences obtained 62,799 unique BLAST hits against the NCBI non-redundant protein data base, including putative orthologs to 8,917 *Drosophila melanogaster* genes based on best reciprocal BLAST hit identity compared with the *D. melanogaster* proteome. Finally, we explored the utility of the transcriptome for RNA-Seq studies, and showed that this resource can be used as a mapping scaffold to detect differential gene expression in different cDNA libraries. This resource will therefore provide a platform for future genomic, gene expression and functional approaches using *P. tepidariorum*.

## Introduction

Comparative studies of animal development can reveal both conserved and derived aspects of developmental mechanisms, providing insight into the developmental basis of evolutionary change of morphology and physiology (“evo-devo”). Many evo-devo studies have focused on arthropods, the most speciose and diverse animal phylum. The largest sub-group of arthropods are the insects (e.g. flies, beetles) with approximately one million described species [1], [2]. Together with the remaining sub-groups (myriapods, crustaceans and chelicerates) the arthropods as a whole comprise almost two thirds of all species on Earth [3].

To facilitate evo-devo research, a broader range of organisms than those traditionally used for laboratory work (often termed "emerging model" species), have been and continue to be established. Despite significant recent advances in gene expression and gene function methodology, the discovery of new and lineage specific developmental genes in emerging model organisms remains a bottleneck due to the difficulty of forward genetic screens and paucity of sequenced genomes for many such organisms. However, the advent and development of Next Generation Sequencing (NGS) technologies now offers solutions to this problem. NGS has been applied to sequence the transcriptomes and genomes of a number of emerging model arthropods. The majority of these are insects with 139 assembled genomes and more than 12 million DNA/RNA and EST sequences publicly available (National Center for Biotechnology Information, NCBI, search term “insecta”, as of February, 2014). For crustaceans three assembled genomes and approximately 2 million DNA/RNA and EST sequences have been submitted to NCBI, while for myriapods only one genome sequence and less than 15.000 DNA/RNA and EST sequences can be found in the NCBI database (NCBI, search terms “crustacea” and “myriapoda”, as of February, 2014). For the chelicerates, genomics and transcriptomics resources are available for several subgroups including horseshoe crabs (Xiphosura), mites (Acari), scorpions (Scorpiones) and spiders (Araneae) (Table S1). Two acari genomes (*Tetranychus urticae*; [4] and *Ixodes scapularis*; [5], [6]), one horseshoe crab genome (*Limulus polyphemus*; [7]) and one scorpion genome (*Mesobuthus martensii*; [8]) have been published to date. Recently genome assemblies for the African social velvet spider *Stegodyphus mimosarum* and the mygalomorph Brazilian white-knee tarantula *Acanthoscurria geniculata* have been published [9].

Spiders have long been the subject of applied biological research aimed at elucidating toxin components [10], the biochemical basis of silk production [11], myosin assembly and muscle function [12], and hemocyte antimicrobial substances [13], [14]. In this context several transcriptomic resources have been generated for spider venom glands [9], [15], [16], silk glands [9], [17], [18], leg muscles [19] and hemocytes [13]. Additionaly, the recently published genome sequences of two spider species and the accompanied transcriptomic data were mainly analyzed in detail with respect to venom and silk genes [9]. Although these resources allow the identification of new genes involved in a given process, they all have in common that they are restricted to adult individuals and in some cases even to a specific organ or tissue (Table S1). Limited embryonic transcriptomic data is only available from the common house spider *Parasteatoda tepidariorum* (previously placed in the genus *Achaearanea*) [20], [21]. However, these data comprise an incomplete set of EST sequences from a single embryonic stage. There is thus a need for a reference transcriptome with comprehensive coverage of multiple embryonic, post-embryonic, larval and adult stages of both sexes of a spider.

Research on spider developmental genetics has mainly focused on two species: the Central American wandering spider *Cupiennius salei* and the common house spider *Parasteatoda tepidariorum*. These efforts have resulted in valuable insight into spider developmental biology and its relation to the spider body plan [22], [23]. Additionally, the basally branching position of chelicerates (including spiders) in the arthropod phylogenetic tree suggests that many findings in spiders will help to infer which developmental processes might be ancestral in arthropods and which ones are derived for a given group.

Two major reasons for the emergence and success of *P. tepidariorum* as the primary chelicerate model for developmental genetics research are its small size and short life cycle. Additionally, a number of essential methods have been established to investigate gene function [23]. Detailed embryonic and postembryonic staging tables allow for exact descriptions of developmental processes [24]–[26] and protocols for immunocytochemistry and whole-mount *in situ* hybridization are available to study cell proliferation, cell death and gene expression patterns [24], [27], [28]. Gene function can be studied by parental or embryonic RNA interference (RNAi) and recent advances include single cell injections that can be used to label and track cell clones or to achieve local misexpression or downregulation of genes [20], [29].

Despite these advances, most recent studies have been constrained by the lack of comprehensive genomic or transcriptomic resources for *P. tepidariorum*. In the absence of such data, the study of genes involved in spider development has relied on the cloning of candidate genes hypothesized to be involved in the process of choice based on studies in model systems like *Drosophila melanogaster*. This leads to a bias towards more conserved genes and makes the discovery of new genes and novel regulatory interactions difficult. In order to overcome this candidate gene approach and to facilitate gene discovery in spiders, we have generated a reference transcriptome for *P. tepidariorum* that encompasses all developmental stages from embryonic to larval, nymphal and adult, and that aims to contain the majority of all mRNAs expressed from the *P. tepidariorum* genome during these stages.

## Results and Discussion

### Sequencing and *de novo* assembly

**Figure 1:**
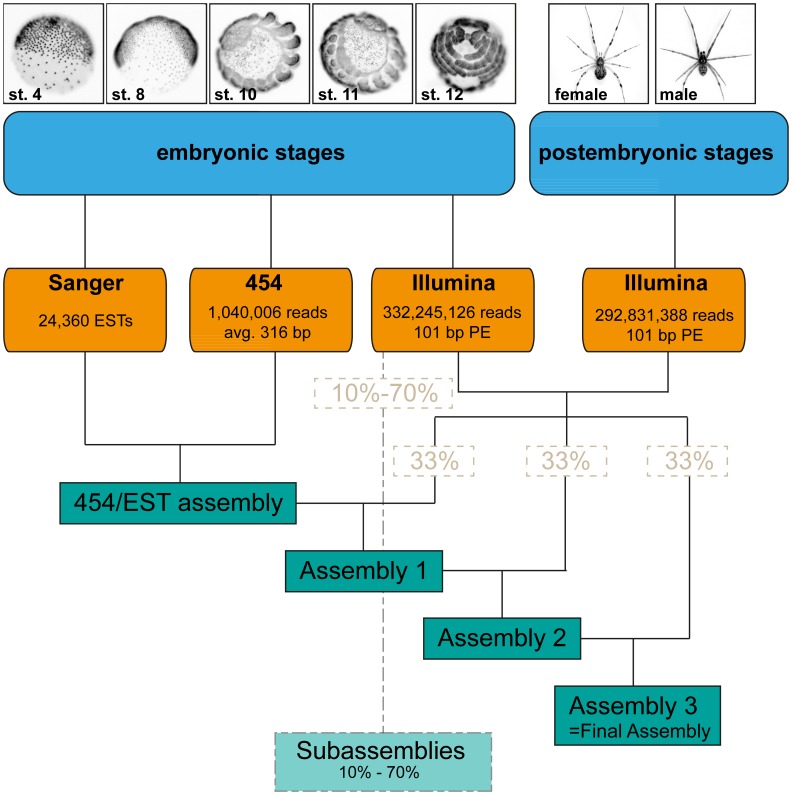
Overview of the sequencing and assembly strategy. Top: Representative stages used for RNA extraction and subsequent sequencing. Bottom: Schematic overview of the sequencing and assembly strategy.

For *de novo* assembly of a comprehensive *P. tepidariorum* transcriptome we used RNA extracted from all embryonic and postembryonic stages, including adult male and female specimens. We combined sequence data generated by various sequencing methods, namely conventional Sanger sequencing (ESTs), Roche 454 pyrosequencing technology, and the Illumina Hi-Seq 2000 platform (see overview in Fig. 1).

**Figure 2:**
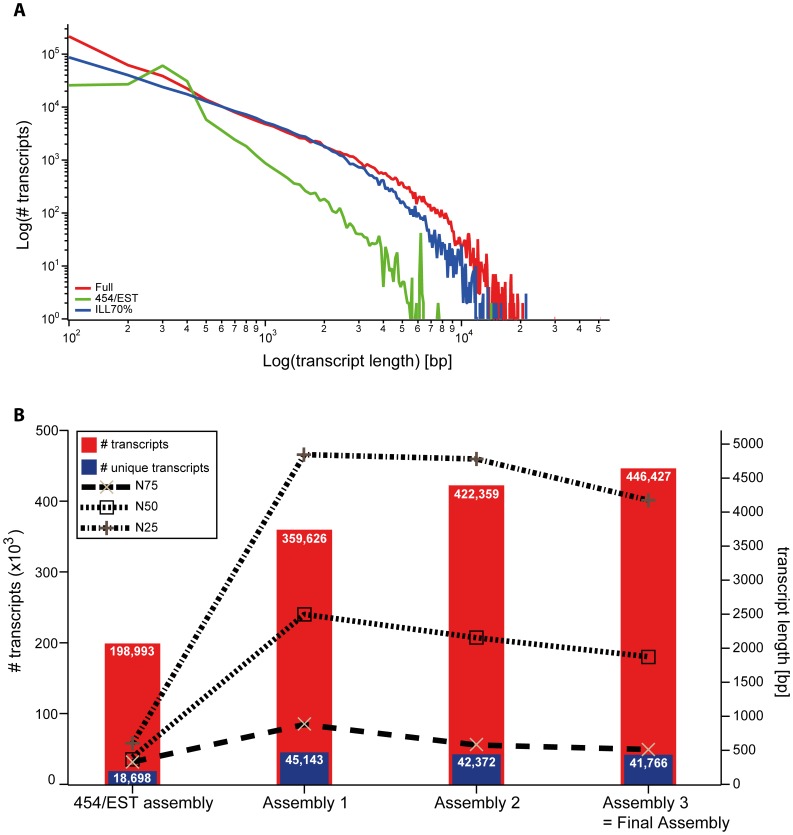
Quality comparison of the 454/EST assembly and the combined final assembly. A Transcript length distribution of different assemblies. Illumina-based assemblies result in more long transcripts compared to the 454/EST assembly. B The 454/EST assembly results in fewer transcripts and fewer unique transcripts based on BLASTX searches against the nr database compared to Illumina-based assemblies (left y-axis). The combination of datasets results in improved NXX (N25, N50, N75) values for the respective assemblies (right y-axis). Note that Assemblies 1 and 2 are the intermediate assemblies of the stepwise assembly strategy and Assembly 3 is the final assembly including unsupported 454/EST transcripts (see Materials and Methods for details).

To generate an embryonic transcriptome for *Parasteatoda tepidariorum*, we sequenced a cDNA library created from embryonic stages 1–14 (according to [26]) using 454 pyrosequencing. These stages represent all developmental events ranging from the first cleavages (stage 1), germ disc (stage 4) and germ band formation (stage 7) to germ band retraction (stage 12) and dorsal (stage 13) and ventral closure (stage 14) ([26]; Fig. 1). Then using the *de novo* assembler Newbler (version 2.6), the resulting reads (see Table 1) were assembled with 22,812 previously published ESTs ([20], [21]; FY216297-FY225483 and FY368221-FY381845) and 1,548 newly generated ESTs (generated from an embryonic cDNA library covering stages 1 to 13) into 21,989 isotigs and 177,004 singletons (Table 2; Fig. 2B). The N50 for the 454/EST assembly is 398 bp, meaning that 50% of the assembled sequences are 398 bp long or longer (Table 2; Fig. 2B).

**Table 1:** Basic statistics for the raw data used for *de novo* assembly.

	# reads	# bp	mean sequence length [bp]	Minimum length [bp]	Maximum length [bp]	1st quantile [bp]	Median [bp]	3rd quantile [bp]
**Illumina raw data**								
**A**	332,245,126	33,556,757,726	101	101	101	101	101	101
**B**	292,831,388	29,575,970,188	101	101	101	101	101	101
**SUM**	**625,076,514**	**63,132,727,914**						
**Illumina trimmed**								
**A_single**	587,707	45,888,998	78.08±27.79	1	101	56	94	101
**B_single**	310,890	24,417,133	78.54±26.20	1	101	58	92	101
**A_paired**	331,060,788	32,414,902,686	97.91±11.53	1	101	101	101	101
**B_paired**	292,205,142	29,021,347,000	99.32±8.48	1	101	101	101	101
**SUM**	**624,164,527**	**61,506,555,817**						
**(% of raw data)**	**(99.85)**	**(97.42)**						
**454 data**								
**454 raw**	1,040,006	639,294,706	614.70±181.69	53	2,045	516	569	650
**454 trimmed**	**1,030,804**	**316,313,188**	306.86±126.22	20	1,977	242	345	395
**(% of raw reads)**	**(99.12)**	**(49.48)**						
**EST data**								
**NCBI EST**	22,812	15,564,653	682.30±141.27	50	1,302	625	690	778
**unpub. EST**	1,548	972,406	628.17±153.94	156	1,354	538.75	658	745
**SUM**	**24,360**	**16,537,059**						
								
**SUM all data**	**625,219,691**	**61,839,406,064**						

**Table 2:** Basic statistics for the 454/EST assembly and the final comprehensive assembly ( = Full assembly 3).

	454/EST	Full assembly 3
**# contigs ("transcripts")**	21,989	446,427
**# singletons**	177,004	NA
**N25 [bp]**	597	4,174
**N50 [bp]**	398	1,875
**N75 [bp]**	329	512
**# transcripts with nr BLAST hits (% of all transcripts)** *	20,447 (10.28)	235,588 (52.77)
**# unique BLAST hits against nr (% of all transcripts)** *	18,200 (9.15)	62,799 (14.07)
**# unique BLAST hits against ** ***D. melanogaster*** ** (% of all transcripts)**	18,698 (9.4)^#^	41,766 (9.36)+
**# orthologs identified by gene prediction (incl. putative paralogs)**	6,255	8,917
**# orthologs identified by gene prediction (w/o putative paralogs)**	5,554	6,396

+ : e-value cutoff = 1e^−10^

*,^#^: e-value cutoff = 1e^−5^

Additional data were obtained by sequencing one embryonic (stage 1–14) and one postembryonic (nymph stages, adult male and female) cDNA library on two lanes of the Illumina HiSeq2000 platform, resulting in 332,245,126 embryonic and 292,831,388 postembryonic paired-end reads of 101 bp length each. To assemble the full transcriptome of *P. tepidariorum* as completely as possible (i.e. attempting to identify and fully sequence all existing transcripts from all life cycle stages) quality trimmed reads of the two Illumina libraries (Table 1) were combined with the isotigs and singletons from the previous 454/EST assembly into a total of 366,721 transcripts using Velvet (version 1.2.08) and Oases (version 0.2.08) [30], [31].

We found that 79,706 sequences longer than 110 bp originating from the 454/EST assembly ended up in the unused reads fraction of the Velvet/Oases assembly. We suspected that those 454/EST based transcripts were likely to represent embryonic transcripts or fragments thereof that did not pass the coverage cutoff set (-cov_cutoff 3) applied for the assembly. This could be due to a low number of Illumina reads supporting these long fragments, as well as to a low abundance of these long sequences itself. One further reason for the discrepancy between the 454/EST sequence set and the Illumina sequence set might be differences in library preparation and fundamental differences in processes like immobilization, cluster formation, elongation and imaging between the two sequencing platforms that we used [32]. In a study by Luo et al. (2012) [33], more than 10% of all 454 reads were shown to be platform specific when compared to Illumina reads obtained from the same DNA sample, further supporting the hypothesis that the differences in the results obtained using the two technologies is due to platform-specific biases. Since it is likely that the almost 80,000 454/EST sequences represent real transcripts, these sequences were incorporated into the full assembly after the Velvet/Oases run, resulting in 446,427 transcripts composed of 274,224,393 bp with a N50 of 1875 bp (Table 2; Fig. 2B). Apart from the 79,706 454/EST-only sequences, a number of Illumina reads also ended up in the unused reads fraction. In total, 171,286 high-quality Illumina reads were not incorporated into the final Velvet/Oases assembly. Since these reads were all shorter than 100 bp, they were omitted from the final assembly (termed "Assembly 3" in Fig. 1). All transcripts of the final comprehensive assembly are accessible via the Assembled Searchable Giant Arthropod Read Database (ASGARD) ([34]; http://asgard.rc.fas.harvard.edu).

### GC content and alternative splicing

The GC content of predicted open reading frames (see Materials and Methods) of the final assembly is 35.9%, which is a relatively low value compared to the coding sequence GC content of other arthropod genomes analyzed to date. These include the first 12 sequenced *Drosophila* genomes (47–55%, [35]), as well as those of *Apis mellifera* (39%), *Nasonia vitripennis*, *Tribolium castaneum* (46%), *Acyrthosiphon pisum* (38%), *Daphnia duplex* (47%) [36], [37], *Mesobuthus martensii* (42.7%) [8] and *Tetranychus urticae* (37.8%) [4]. The observed GC content is also low in comparison to estimates for coding sequences of various vertebrates like *Danio rerio* (47.9%), *Xenopus laevis* (48.1%), *Mus musculus* (53.2%) and *Gallus gallus* (55.1%) [38]. The significance of the low GC content of *P. tepidariorum* found in the transcriptome data is unclear. Generally it has been shown that coding regions are more GC-rich compared to surrounding genomic regions [39], [40]. If this is true for *P. tepidariorum*, the GC content of the entire genome is expected to be much lower than the 35% observed for this transcriptome. A GC content of 30.3% for the entire final assembly including untranslated regions and potential non-coding RNAs supports this hypothesis. The GC content of coding sequences has also been shown to correlate positively with transcript length [41] and expression level of the respective locus [42], suggesting that *P. tepidariorum* transcripts might tend to be shorter and that overall transcription activity is lower compared to other species.

Many *de novo* assembly algorithms attempt to predict alternatively spliced isoforms of a given transcript. As part of the Oases step, sequences that share high similarity are pooled into so-called loci. The transcripts within one locus ideally represent all RNA isoforms of the same gene [31]. In order to estimate the accuracy of this clustering process, we analyzed the number and composition of loci in the final assembly. The 446,427 transcripts of the final assembly were clustered into 106,589 loci with on average 1.9 transcripts per locus. However, we observed that locus 1 encompasses 166,847 transcripts of various lengths and with very different gene predictions, showing that the similarity analysis performed by the Oases algorithm has its limitations and tends to combine unrelated transcripts if they do not match other loci defined by the algorithm. This effect might be related to the long-branch attraction artifact known from other similarity analyses that also use phylogenetic criteria for similarity [43]–[45]. Another explanation for the prediction of highly complex loci might be the presence of sequencing errors, because transcripts that only differ by one nucleotide will be treated as two individual sequences of the same cluster. Since the RNA for the different libraries was extracted from various individuals at different time points (see Materials and Methods) it is also possible that naturally occurring polymorphisms contribute to the assembly of complex clusters. However, this effect might only account for a small portion of the complexity since the Göttingen spider culture is inbred since at least 18 generations. Although locus 1 must be regarded as a computational artifact, there are other loci with higher numbers of transcripts that might be real: We found 13 loci with 100 or more transcripts and 3,909 loci with 10 or more transcripts. This data is supported by a recent detailed study of the *Drosophila melanogaster* transcriptome, which shows that 90% of all genes are transcribed into at least 10 transcripts and five isoforms [46]. Brown et al., even identified 47 genes that have the potential to code for more than 1,000 isoforms each, suggesting that highly complex loci as identified in our study might be real. In order to test this hypothesis, we analyzed the locus with the second most transcripts in detail (Locus 1038, 398 transcripts). 81.4% (324 of 398 transcripts) of these transcripts are shorter than 500 bp. Of these only 23 transcripts (7.1%) resulted in BLAST hits against nr. Of those transcripts longer than 500 bp (18.6%) nearly 50% (36 of 74 transcripts) resulted in a BLAST hit. In total, 14.8% (59 of 398 transcripts) resulted in a BLAST hit against nr with 22 different hits. The other twelve loci with more than 100 transcripts show a similar pattern with a high number of short transcripts and 3 to 25 different BLAST hits per locus (not shown). On one hand this data suggests that these loci are very complex because of the inclusion of many short transcripts. On the other hand, based on BLAST annotations, these clusters are likely to represent at least in part artificial clustering of transcripts. One general approach to reduce the complexity of the assembly for subsequent analyses is to retain only one transcript per locus [47]. However, if this method had been applied to the present assembly, over 166,000 transcripts would have been lost. Since our main aim was to provide a comprehensive transcriptome, we decided to keep all transcripts for further analysis. A complete analysis of isoform complexity will only be possible when the transcriptome data presented here are combined with genome sequences for genome annotation.

### Annotation of the final transcriptome

All transcripts of the final assembly were compared with the NCBI non-redundant protein database (nr) using BLASTX to estimate the number of unique transcripts in the obtained dataset. We found that 52.8% (235,588) of all transcripts (Table 2) showed similarity to at least one protein in the nr database, applying an E-value cutoff of 1e-5. On average 3.4 transcripts of the final assembly share the same hit against nr (maximum: 3,613; minimum: 1). Additionally, it is expected that many transcripts may show similarity to the same nr accession due to sequencing errors and the potential of the assembler to predict different isoforms. Therefore, we obtained the number of distinct nr accessions that mapped to at least one predicted transcript in the final assembly (defined as unique transcripts). This analysis revealed 62,799 unique transcripts (Table 2). A BLASTX search against the *Drosophila melanogaster* proteome (FB2011_03 Dmel Release 5.35; 23,361 protein sequences) with an E-value cutoff of 1e-10 identified 41,766 unique transcripts (Table 2; Fig. 2B). Applying a similar approach, only ∼40,000 unique transcripts were predicted for two spider species based on transcriptome analysis [48]. One reason for this discrepancy might be that this dataset is based on adult spiders, while the transcriptome presented here represents all developmental stages and both sexes. However, based on these results it is of course difficult to estimate the number of potential gene models present in the *P. tepidariorum* genome. The main reason is that in both protein databases used to identify unique transcripts, more than one protein sequence of the same locus might be present. Another source of uncertainty about the true number of gene models in *P. tepidariorum* might be the presence of incompletely assembled transcripts, especially those resulting from incorrectly assembled untranslated regions (UTRs), which could result in false positive BLAST hits. In addition, the high number of transcripts within some of the loci (see above) might artificially increase the apparent complexity of the transcriptome. Altogether, the present transcriptome analysis is likely to represent an overestimation of the actual gene number in genome. For the two recently published spider genomes the predicted number of gene models ranges from 27,235 in the velvet spider to ∼70,000 in the tarantula [9]. Additionally, the estimated number of gene models in other chelicerate species for which genome sequences are available ranges from 18,414 gene models in the spider mite *Tetranychus urticae* [4] and 24,925 gene models in the tick *Ixodes scapularis* [6] (IscaW1.2 gene set) to 32,016 gene models in the genome of the scorpion *Mesobuthus martensii* [8]. Only a thorough analysis of the *P. tepidariorum* genome will reveal a conclusive estimate of the number of gene models present in this species.

**Figure 3:**
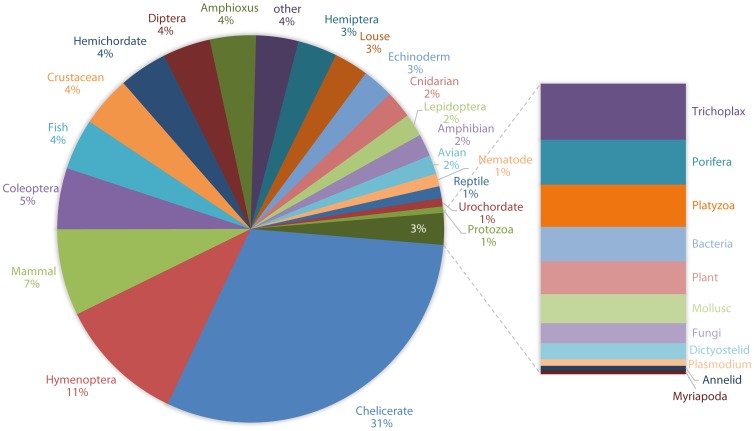
Species distribution of best BLASTX hits against the nr database. Most best BLASTX hits against the non-redundant protein database (NCBI) originate from chelicerate sequences.

We also analyzed the similarity of the assembled spider transcripts to those of other species represented in the nr database. To this end, we counted how often a given taxonomical group occurred as best BLAST hit. The highest proportion of best BLAST hits was found for Chelicerates (31%), then Hymenoptera (11%) and Mammals (7%) (Fig. 3), suggesting a high level of lineage-specific genome evolution in chelicerates. This finding is compatible with the recent discovery of a high number of chelicerate-specific gene families found in the spider mite [4] and scorpion [8] genomes, where a very high turnover rate (gain and loss of gene families) has been observed.

Finally, we further analyzed those transcripts that did not show similarity to any of the protein sequences stored in the nr database. A BLASTX search against the nr database with an E-value cutoff of 1e-10 resulted in 53,247 (11.9%) transcripts with and 393,180 (88.1%) transcripts without a BLAST hit respectively (Fig. S1A). Both sets of transcripts were queried against the PROSITE functional protein domain database [49], [50] using an InterPro scan [51], [52]. This analysis suggested that 2,674 transcripts that did not have BLAST hits against nr are nevertheless likely to code for functional proteins (Fig. S1A). A comparison of these protein domains to those predicted for transcripts that showed BLAST hits against nr did not reveal a qualitative difference in the protein domains found in both datasets (Fig. S1; Table S2). In both groups, the Zinc Finger C2H2 domain was the most abundant domain followed by the Protein Kinase ATP and Protein Kinase ST domains (Fig. S1B; Table S2).

The high number of transcripts that neither showed sequence similarity to sequences present in the nr database, nor seemed to code for functional protein domains, might be due to the high number of short transcripts (100–500 bp) in the final assembly. Of the more than 440,000 transcripts in the final assembly, 75.8% (338,487 of 446,427) are shorter than 500 bp (see also below). Only 5.7% of those short transcripts show BLAST hits against nr, while 46.2% of the transcripts longer than 500 bp result in hits against the nr database. This data is further supported by ESTSCAN (v-3.0.3) results showing that only 10.8% of the short transcripts have coding potential. In contrast, 49.6% of the longer transcripts (>500 bp) code at least partially for proteins or peptides. A similar result has been shown for other spider transcriptomes, where 30–40% of transcripts longer than 200 bp could be annotated based on BLAST searches against various protein databases [18], [48]. Furthermore, it is likely that many of the transcripts with no clear BLAST hit against nr might represent non-coding RNAs since only 2.4% (9,052 of 377,291) and 8.6% (32,304 of 377,291) of these sequences show coding potential based on open reading frame prediction and an ESTSCAN, respectively. In contrast, 71.3% (49,279 of 69,136) and 83.8% (57,907 of 69,136) of the transcripts with BLAST hits against nr have open reading frames or at least ESTSCAN results, respectively. It has previously been shown that the expression of a large variety of non-coding RNAs ranging from a few nucleotides (e.g. microRNAs, small interfering RNAs or PIWI-associated RNAs) to several kilobases (e.g. long non-coding RNAs, lncRNAs or X-inactive-specific transcript RNA, Xist RNA) [53] are potential sources of transcribed sequences without clear similarity to other sequences in the database. In *Drosophila melanogaster* a recent transcriptome sequencing study revealed nearly 2,000 potential lncRNAs that code for approximately 3,000 transcripts with no clear protein-coding potential [46]. An alternative source of transcripts with no clear orthology to functional proteins are retroelements, which have been shown to significantly contribute to transcriptomes of mammalians [54], [55] and insects [56]. Using RepeatMasker (version 4.0.5) we identified potential retroelements in the entire final assembly. This analysis revealed that 28% (19,368 of 69,136) and 13,1% (49,413 of 377,291) of the transcripts with and without BLAST hits, respectively were masked, suggesting that retroelements are unlikely to be enriched in those transcripts without clear orthology. And finally, one potential natural source of expressed transcripts lacking sequence and protein domain conservation are small open reading frames (smORFs), which are hard to identify by traditional means of gene prediction [57]. However, it has been demonstrated that the small peptides translated from smORFs can play important roles during development [58]–[61] and for proper adult organ function [62].

### Identification of *D. melanogaster* orthologs by gene prediction and developmental pathway analysis

**Figure 4:**
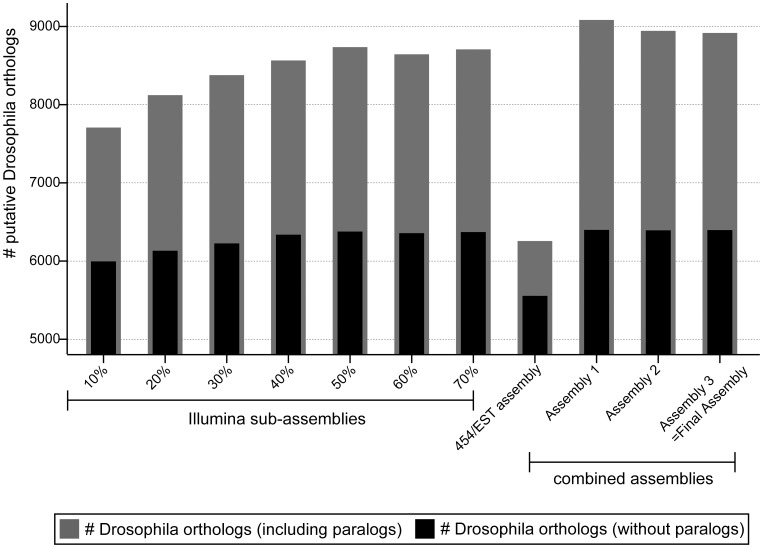
Comparison of gene prediction results for different assemblies. Gene prediction is based on reciprocal BLASTX hits against the *D. melanogaster* proteome. The number of *D. melanogaster* orthologs with or without paralogs is shown for Illumina-only assemblies, the 454/EST assembly and the three combined assemblies. The use of Illumina data results in the identification of more orthologs compared to the 454/EST assembly.

In order to obtain high-confidence gene predictions we annotated the transcriptome applying a previously developed automated annotation pipeline, which is based on best reciprocal BLAST hits against the *D. melanogaster* proteome with an E-value cutoff of 1e-5 [34], [63]. This method of gene prediction resulted in the identification of 8,917 putative *Drosophila* orthologs. This set of predictions includes *P. tepidariorum* transcripts with a top BLAST hit being a *D. melanogaster* gene that does not have it as reciprocal top BLAST hit. We defined these as paralogs. If paralogs are excluded, the number of predicted *D. melanogaster* orthologs is reduced to 6,396 (Table 2; Fig. 4). These data show that our transcriptome represents an 8416% improvement in terms of annotated sequence availability for *P. tepidariorum,* given that only 76 such mRNA sequences were present in the NCBI database (as of January, 24^th^ 2014). The predicted putative orthologs for all transcripts are stored in the ASGARD database, where all sequences and gene prediction information can be freely accessed ([34]; http://asgard.rc.fas.harvard.edu).

**Figure 5:**
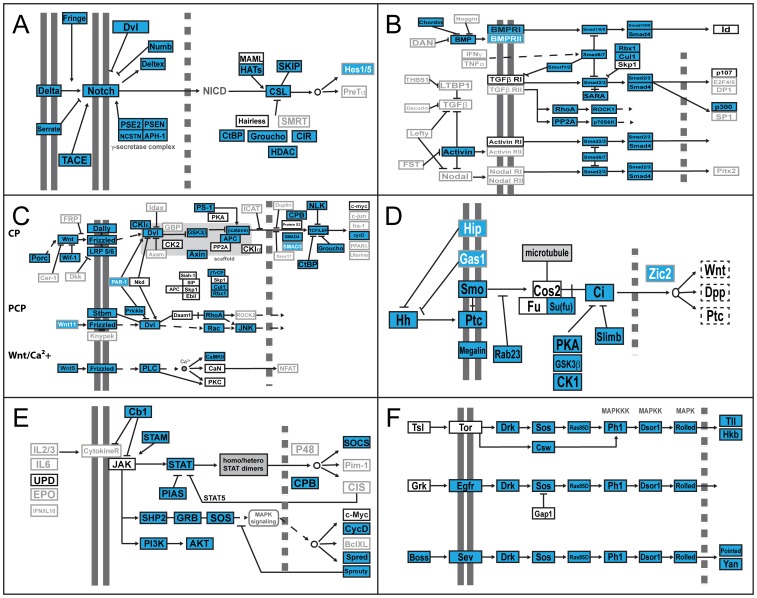
Analysis of conserved developmental signaling pathways. Pathway components were extracted from the KEGG database (http://www.genome.jp/kegg/kegg1.html). Sequences mainly from *D. melanogaster* were compared to the final *P. tepidariorum* assembly by BLAST. In cases where no *D. melanogaster* ortholog was present (grey boxes with grey or white typeface) the respective mouse or human sequence was used as query (see Table S3 for details). Genes identified in the spider transcriptome are marked in blue. Pathway schematics modified from KEGG pathway model images http://www.genome.jp/kegg/kegg1.html. A Notch pathway; B TGFβ pathway; C Wnt pathway; D Hedgehog pathway; E Janus Kinase (JAK)-signal transducer and activator of transcription (STAT) pathway; F Mitogen-activated protein Kinase (MAPK) pathway.

We used this information about confidently identified and annotated transcripts to explore the presence of components of important developmental pathways (Fig. 5; Table 3; Table S3). Specifically, we searched for a total of 208 pathway components on the basis of previous knowledge of their role in these pathways in *Drosophila*, mammals or other animal models (based on the KEGG database [64], [65]). We found that more than 60% of these preselected pathway components were also present in the *P. tepidariorum* transcriptome. Sequence information for only 16.8% of those had been published and made available previously (as of January, 24^th^ 2014) (Table 3).

**Table 3:** Analysis of developmental pathways.

	# pathway components	# present (% of total)	# absent (% of total)	# previously known(% of present)
**Delta/Notch**	25	21 (84)	4 (16)	4 (19.05)
**TGF beta**	47	23 (48.94)	24 (51.06)	2 (8.7)
**WNT**	75	45 (60)	30 (40)	12 (26.67)
**Hedgehog**	16	14 (87.5)	2 (12.5)	4 (28.57)
**JAK/STAT**	27	14 (51.85)	13 (48.15)	0
**MAPK**	18	14 (77.8)	4 (22.2)	0
	**208**	**131 (62.98)**	**77 (37.02)**	**22 (16.79)**

Pathway components are based on the KEGG database.

For some of the pathways we found that the *P. tepidariorum* transcriptome contains putative orthologs of more than 80% of all known components, as in the case with the Delta/Notch (Fig. 5A, Table 3) and Hedgehog pathways (Fig. 5D, Table 3). This suggests that the level of lineage-specific gene loss is low for those pathways. Indeed, in other arthropods, like the pea aphid *Acyrthosiphon pisum* [66], the red flour beetle *Tribolium castaneum* [67], [68] and the crustacean *Parhyale hawaiensis* [69], these two pathways also contain orthologs of nearly all known components based on genome and transcriptome data. In addition, for the Hedgehog pathway, we identified a putative ortholog of the Hedgehog Interacting Protein (Hip) that had not been found outside of vertebrates to date (KEGG database).

Of the mitogen-activated protein (MAP) Kinase pathway members (Fig. 5F, Table 3) only the tyrosine-protein kinase receptor Torso (Tor), the torso-like protein (Tsl) and the EGFR ligand Gurken (Grk) were not found in our dataset. The absence of Grk is consistent with its known absence from the genomes of the red flour beetle [67] and the pea aphid [66], suggesting that this ligand might be a *Drosophila*-specific factor. However, we identified at least one putative Spitz/Keren-like ortholog (not shown), which might play the role of an EGFR ligand in *P. tepidariorum*.

More than 50% of all pathway components of the Janus kinase/Signal Transducer and Activator of Transcription (JAK/STAT) pathway as defined in the KEGG database have putative orthologs in our spider transcriptome (Fig. 5E; Table 3). However, inconsistent with the tick *Ixodes scapularis* (KEGG database) and most insects except for *Drosophila* [66], [67], we did not find an ortholog of the JAK/STAT pathway ligand Unpaired. In fact, we did not identify any clear JAK ortholog in the spider transcriptome, although an ortholog has been described in insects, in a crustacean and in the tick ([66], [67], [69]; KEGG database) (Fig. 5E, Table 3).

All core components of the canonical Wnt pathway, the planar cell polarity pathway and the Wnt/Ca^2+^ pathway were present in the spider transcriptome (Fig. 5C; Table 3; Table S3). In addition to a large repertoire of Wnt ligands (discussed extensively elsewhere, [70]), we found one additional Frizzled receptor in the spider (Table S3).

Based on gene prediction we could identify only about half of the Transforming Growth Factor beta (TGFbeta) pathway component orthologs in the spider transcriptome (Fig. 5F, Table 3; Table S3). While most intracellular components were present, receptors and extracellular components were largely missing from our dataset. For instance, we only found one group of putative receptors (BMP), while TGFbeta, Activin and Nodal receptors were missing. In the spider mite, six conserved TGFbeta/activin receptor members have been identified [4] and in most insects and in the crustacean *Parhyale hawaiensis* at least one TGFbeta/activin receptor ortholog is present (KEGG database; [69]).

### Quality assessment

#### Estimation of sequencing depth

To estimate the sequencing depth in our stepwise assembled transcriptome (see Materials and Methods, Fig. 1), we mapped all Illumina reads to the final assembly and counted the number of reads per transcript. Only 8,920 (2%) of the 446,427 sequences in the final transcriptome did not have matching Illumina reads, implying that these sequences must be derived from the 454 or EST sources. 5,555 (1.24%) transcripts had only one read mapped, while 9,142 (2.05%) transcripts were covered by more than 10,000 reads. In general we found a positive but low correlation between transcript length and number of mapped reads (Figure S2; Pearson Correlation: r_446,425_ = 0.13, p<2.2e^−16^; Spearman Correlation: rs = 0.65, N = 446,427, p<2.2e^−16^). In order to account for different transcript lengths, we calculated coverage as the number of reads divided by the length of the respective transcript. In this length-normalized dataset the mean coverage per transcript was 2.8 reads/bp. 24.68% of all transcripts (110,176 of 446,427) showed a coverage of at least 1 read/bp and 21.53% of all transcripts (96,095 of 446,427) showed a coverage between 1 and 10 reads/bp, while the coverage for 1,569 transcripts (0.35%) exceeded 100 reads/bp (Figure S3). The coverage estimate presented here is very likely an underestimation of the real coverage since only the Illumina reads were considered. Due to the mix of different read types and sequencing technologies, it is impossible to compare the per transcript coverage to previous single-platform based transcriptome assemblies.

#### Estimation of completeness of the transcriptome

We also asked if the level of gene discovery in our final assembly was likely to be saturated. To test whether more reads also lead to a more complete transcriptome assembly in terms of identified transcript species, we used randomly sampled progressively larger subsets of reads originating from one of the Illumina lanes (Sample A, embryonic), which were assembled *de novo*. The resulting transcripts were annotated by BLASTX searches against the *D. melanogaster* proteome and gene prediction based on reciprocal BLAST hits against the genome of *D. melanogaster*.

**Figure 6:**
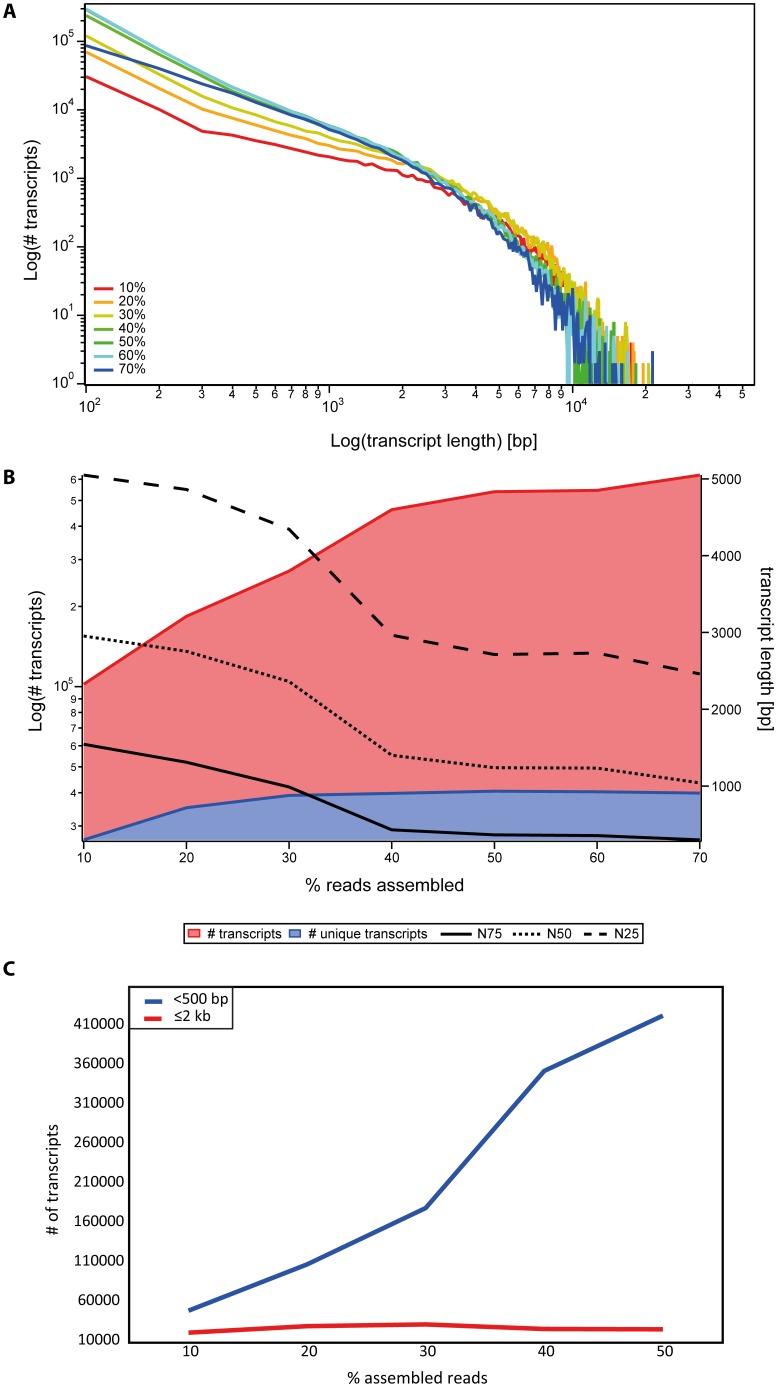
Estimation of transcripome completeness. Progressively larger subsets of reads (10%–70%) originating from one Illumina lane (embryonic sample, Sample A) were assembled *de novo*. A The use of more reads resulted in the assembly of very long transcripts, but more short transcripts were observed. B Using more Illumina reads allowed the assembly of more transcripts. The number of unique transcripts does not increase if more than 40% of the reads of one Illumina lane are used. Similarly, the NXX values of the subassemblies where more than 30%–40% of the reads were assembled are lower due to the high number of short transcripts. C The number of transcripts shorter than 500 bp increases much more than the number of transcripts longer than 2 kb if more reads are assembled.

As expected, the use of an increasing number of reads in the *de novo* assembly resulted in a growing number of transcripts (Fig. 6B, red graph). While 10% of the reads of one Illumina HiSeq2000 lane could be assembled into roughly 100,000 transcripts, the use of 70% of one lane yielded six times as many transcripts, and the number of transcripts did not seem to have reached a plateau (Fig. 6B, red graph). However, the analysis of transcript lengths shows that the addition of more reads mainly led to an increase of relatively short transcripts (100–500 bp), but had no obvious influence on the longer transcripts (Fig. 6A, C). This was also mirrored by a reduction in the N50 value if more than 30% of the reads were used in the assembly (Fig. 6B). In fact, although the total number of transcripts increases over all subsets, the number of unique BLASTX hits against the *D. melanogaster* proteome reached a plateau at approximately 40% of available reads (Fig. 6B, blue graph). Similarly, the gene prediction pipeline did not reveal more *D. melanogaster* orthologs once more than 40–50% reads from one Illumina lane had been assembled (Fig. 4). This indicates that most of the additional transcripts obtained when more than 40% of the reads were used, do not represent additional transcript species, but rather are likely to be small non-overlapping parts of transcript species already accounted for in the assembly.

In summary, the assembly and subsequent annotation of progressively larger datasets of Illumina reads suggests that gene discovery was saturated when 30–50% of the reads were used. In principle, therefore, half an Illumina HiSeq2000 lane might be sufficient to identify most of the transcript species (but not their complete sequence: see also below) in a gene-rich transcriptome like the one of *P. tepidariorum* presented here.

#### Multiplatform assembly results in recovery of longer and more unique transcripts

We compared the length distribution of assembled transcripts of the 454/EST assembly and an assembly based on only high quality Illumina reads (the 70% assembly of Sample A, see above) to the combined final assembly. All three approaches resulted in comparable numbers of transcripts with a length of up to 400 bp (Fig. 2A). Using the Illumina data alone allowed the assembly of longer transcripts (up to 21 kb) and increased the number of transcripts longer than 400 bp (Fig. 2A). This suggests that, with our assembly method, the numerous short reads produced by the Illumina technology are superior to the longer but fewer reads produced by Roche 454 with respect to assembled transcript length. However, when we combined both datasets, we not only obtained the highest number of long transcripts, but also the longest transcripts of all datasets.

We cannot rule out that this positive effect of combining data from both sequencing platforms was caused by artificial transcript fusions (i.e. computational merging of unrelated sequences into longer but false "transcripts"). However, based on gene prediction (against *D. melanogaster*) and BLAST searches against nr, these long transcripts indeed have orthologs. For instance, the longest transcript in the whole survey (55.9 kb) shows high similarity to vertebrate TOR proteins including the conserved kinase domain (not shown). Additionally, it is likely that very long transcripts do exist in the *P. tepidariorum* transcriptome, since several extremely long coding sequences have already been described in the genomes of other organisms. For example, the human *titin* gene, which is involved in building muscle structure, has a coding sequence of 82 kb [71].

The combination of Illumina data and the combined 454/EST assembly not only resulted in longer transcripts, but it also improved the overall assembly quality as measured by N50 values and in terms of annotation. While the N50 of the 454/EST assembly alone was only 398 bp, the sequential addition of Illumina data resulted in an N50 value of 1875 bp (Fig. 2B). BLASTX searches against the *D. melanogaster* proteome resulted in more unique transcripts when the 454/EST data was combined with Illumina reads (Fig. 2B). Finally, the gene prediction pipeline based on reciprocal BLAST hits identified 42.56% more *D. melanogaster* orthologs (15.16% if paralogs are excluded) when the data from different sequencing platforms was combined during the assembly procedure (Table 2; Fig. 4).

In summary, we could show that with our assembly method the use of Illumina reads for *de novo* transcriptome assembly clearly outperforms the 454 pyrosequencing platform alone. This is very likely due to the much higher throughput of the Illumina HiSeq2000 technology, which can produce up to 200 Gb per run, while the 454 FLX Titanium platform can generate 0.5 Gb per run [72]. However, our data also demonstrate that the longer average read length obtained with 454 sequencing (more than 600 bp; Table 1) in combination with a high number of shorter Illumina reads facilitates the assembly of longer transcripts.

#### Estimation of completeness of assembled transcripts

**Figure 7:**
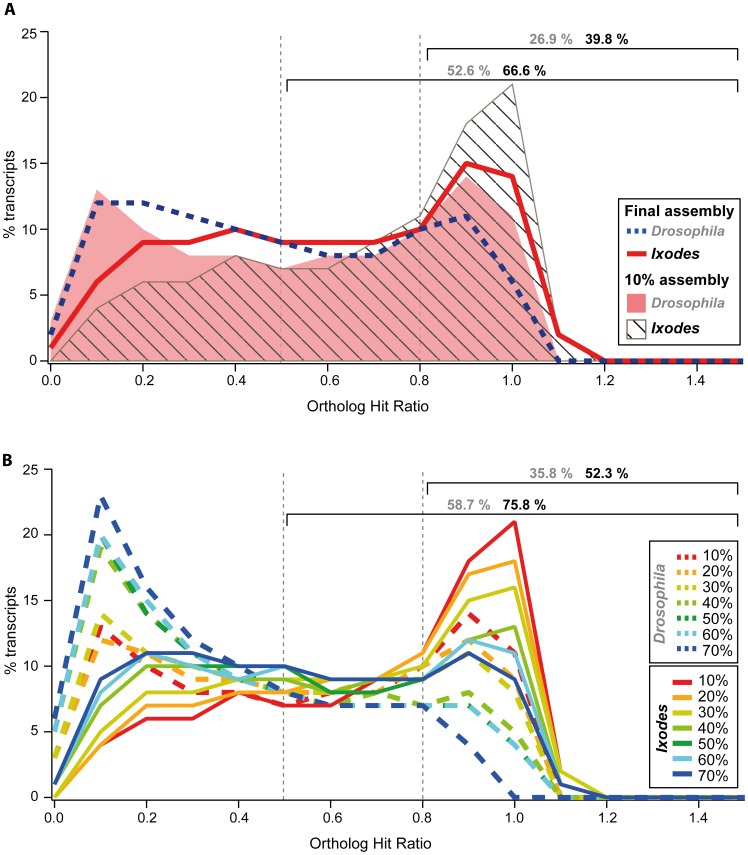
Ortholog hit ratio (OHR) analysis of assembled transcripts in the *P. tepidariorum* transcriptome. An OHR of one suggests that a transcript has been assembled to its true full length. BLAST was used to compare the final spider assembly with the *D. melanogaster* and the *I. scapularis* gene set, respectively. A The OHR analysis for final assembly shows that more than 25% and nearly 40% of all transcripts have an OHR of 0.8 compared to *Drosophila* and *Ixodes* respectively. Note that the percentages given above the brackets are based on the final assembly. The data for the 10% subassembly is shown only as comparison. B The OHR analysis shows that the use of more Illumina reads for the assembly resulted in more transcripts with low OHR. This effect is due to the assembly of more very short transcripts (see also Fig. 6). Note that the percentages given above the brackets are based on the 10% subassembly.

After assessing the assembly quality based on transcript length and gene discovery, we investigated to what degree the final assembled transcripts might represent real full-length sequences. One way to estimate this is to calculate the ortholog hit ratio (OHR) [73]. This value is calculated as the ratio of the number of continuous homologous nucleotides of a *de novo* assembled transcript and the total length of the best ortholog in the proteome. Ideally, the OHR is calculated on the basis of genomic information of the organism of interest. However, since no annotated genome sequence for *P. tepidariorum* is available yet, the OHR was defined based on the proteomes of the fruit fly *D. melanogaster* and the tick *I. scapularis*, respectively. A spider transcript with an OHR of 1 would represent a putative full-length transcript based on comparisons to the fly or tick ortholog sequences. We found that 52.6% of all assembled transcripts represented 50% of the respective *Drosophila* sequence, and 26.9% of all transcripts were likely to represent at least 80% of the predicted full-length sequence (Fig. 7A). A much higher OHR could be observed when *P. tepidariorum* predicted transcripts were compared to *Ixodes* sequences. In this case, 66.6% and 39.8% of all spider transcripts represent 50% and 80% of the given predicted full ortholog length, respectively (Fig. 7A). This result is consistent with the fact that *P. tepidariorum* and *I. scapularis* are phylogenetically more closely related to each other than either species is to *D. melanogaster*. Accordingly, we showed based on BLAST results that there is an overall much higher sequence similarity of the spider transcriptome to chelicerates compared to dipterans (Fig. 3).

Although the proportion of potentially fully assembled transcripts is already high, the OHR values calculated here might represent an underestimation of transcript completeness due to a high number of short transcripts in the final assembly. As discussed above, the enrichment of short transcripts is due to the assembly of a high number of Illumina reads (compare 10% to 70% in Fig. 6A; see also Fig. 6C). In order to estimate the influence of the number of short transcripts on the OHR, we calculated this value for the subassemblies that were based on progressively larger datasets (Fig. 7B). This analysis clearly demonstrates that, consistent with the assembly of more short transcripts, the OHR is lower when more reads are used (compare 10% to 70% in Fig. 6B,C). Consequently, the overall OHR is much higher for the 10% subassembly, where 52.3% (compared to 39.8% for the complete assembly) of all transcripts represent 80% or more of the orthologous *Ixodes* sequences (Fig. 7A). Since many high quality reads of two full Illumina HiSeq2000 lanes were assembled for the final transcriptome presented here, which yielded a proportionally higher amount of short transcripts, it is not unexpected that the OHR is much smaller than expected, although this does not necessarily reflect the quality of the assembly.

### Differential Expression analysis of embryonic and post-embryonic stages

The availability of a comprehensive transcriptomic resource for *P. tepidariorum* opens the possibility to use this transcriptome as a scaffold to map short reads generated in future experiments to study genetic variation and gene expression in this spider. In order to test the general feasibility of such mapping experiments, we used Bowtie2 (version 2.0.2) [74] to separately map the embryonic and post-embryonic reads that were used for the assembly to the final transcriptome.

More than 97% of the high quality and poly-A trimmed reads in both libraries were mapped successfully, with 60% of those reads mapping to more than one position in the transcriptome (Table 4). The high number of successfully mapped reads was expected because the reference was mainly assembled from the reads used for mapping. It is also obvious that many reads were mapped to multiple transcripts in the reference transcriptome because the assembly produces several similar sequences, which very likely represent isoforms of the same gene or paralogous genes with high similarity.

**Table 4:** Basic statistics of the mapping experiment, differential expression analysis and BLAST annotation of enriched transcripts.

	Embryonic Samples	Postembryonic samples
**# raw reads**	332,245,126	292,831,388
**# raw base bairs**	33,556,757,726	29,575,970,188
**# trimmed reads**	331,060,788	292,205,142
**(% of raw reads)**	(99.64)	(99.79)
**# mapped reads**	322,037,900	285,150,827
**(% of trimmed reads)**	(97.27)	(97.59)
**# reads aligned concordantly 0 times**	112,476,108	92,182,498
**(% of trimmed reads)**	(33.97)	(31.55)
**# reads aligned concordantly exactly 1 time**	18,959,642	23,955,144
**(% of trimmed reads)**	(5.73)	(8.20)
**# reads aligned concordantly >1**	199,625,038	176,067,500
**times (% of trimmed reads)**	(60.30)	(60.25)
**# reads aligned discordantly 1 time**	1,096,510	997,018
**(% of reads aligned concordantly 0 times)**	(0.97)	(1.08)
**# enriched transcripts**	29	918
**# enriched transcripts with BLAST hits**	7	349

Using SAMtools [75] we extracted the raw read counts for each transcript in the reference transcriptome for each of the two libraries. After accounting for slight differences in the number of reads used for mapping, the sequences of transcripts that were specific for embryonic and post-embryonic stages respectively were extracted and annotated using Blast2GO [76], [77] (see Materials and Methods for details). We identified only 29 transcripts that were exclusively present in the embryonic sample (with “exclusively present” being the criteria that there were zero reads in the post-embryonic sample and at least 1000 reads in the embryonic sample), while 918 transcripts were exclusively present in the postembryonic sample (using the same criteria for “exclusively present” defined above) (Table 4). This finding suggests that for this pilot analysis, most of the genes that are activated during embryonic development continue to be expressed during post-embryonic stages. Since many developmental factors like HOX genes and components of the Wnt pathway are also required for late differentiation processes and the maintenance of cellular integrity [78], [79] it is possible that many *P. tepidariorum* embryonic genes retain their expression for roles during later development and in adults. Our analysis, however, does not allow quantification of changes in the expression level of those genes during the transition from embryonic to post-embryonic stages.

**Figure 8:**
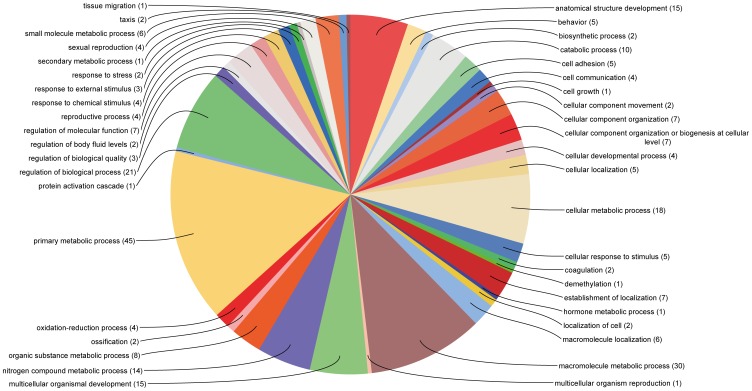
Distribution of gene ontology (GO) terms of transcripts enriched in postembryonic stages. The distribution of GO terms of transcripts that are supported by at least 1000 reads, but have no mapped reads originating from the embryonic sample is shown for Biological Process (Level 3). See also Table S4 for more details. The number of transcripts within the respective category is given in brackets.

The fact that we are able to identify more than 900 transcripts that appear to become activated in post-embryonic stages demonstrates that our comprehensive reference transcriptome is suitable for subsequent mapping approaches. The functional annotation of transcripts that are exclusively present in embryonic and post-embryonic stages resulted in seven and 349 sequences with BLAST hits respectively. Since the Gene Ontology (GO) category analysis for the low number of embryonically expressed transcripts was not informative (Table S4), we performed GO analysis on those transcripts present in post-embryonic stages. Approximately 60% of all post-embryonically expressed transcripts were associated with metabolic processes, response to stimulus and reproduction (Fig. 8; Table S5). 12% of the transcripts were involved in developmental processes (Fig. 8; Table S5), consistent with the fact that our post-embryonic transcriptome was also comprised of early postembryonic stages, which would still have been in the process of extensive morphogenesis and growth.

In summary, we successfully mapped Illumina reads against the reference transcriptome to identify transcripts specifically expressed in post-embryonic stages of *P. tepidariorum*. The GO annotation suggests that these transcripts are functional in post-embryonic biological processes. The fact that we obtained this clear result with a relatively basic experimental setup (two very broadly mixed developmental stages, one replicate) strongly suggests that future RNAseq experiments will profit from this reference transcriptome.

## Materials and Methods

### Tissue samples and RNA extraction

All animals used in this study originate from the Göttingen culture that was inbred for at least 18 generations (inbred since 2008; 4–5 months per generation). For the 454 Pyrosequencing, two embryonic samples (stages 1–5 and stages 6–14 respectively) (staging after [26]) were flash frozen and shipped to Vertis Biotechnologie AG (Freising, Germany) for RNA isolation using mirVana miRNA isolation kit (Ambion).

For high coverage Illumina sequencing two individual samples were prepared from the Göttingen culture as follows: one embryonic sample (Sample A) composed of embryonic stages 1–14 and one post-embryonic sample (Sample B) composed of nymph stages, one adult male and one adult female. For the embryonic sample, 3–5 embryos for each of the early stages (1–5) and 1–2 embryos for each of the later stages (6–14) were used for RNA extraction. All embryos of the same stage were isolated from the same cocoon. All embryonic and post-embryonic stages were pooled respectively and total RNA for both pooled samples was extracted using the RNeasy Mini Kit (Qiagen). The quantity of the extracted RNA was controlled by concentration measurements using the fluorescent dye-based Qubit technology (Invitrogen) and the quality was confirmed by Agilent 2100 Bioanalyzer runs (Agilent Technologies).

### Library preparation and sequencing

The ESTs were sequenced from miniprepped bacterial clones from a pBluescript II SK+ cDNA library generated by Vertis Biotechnologie AG (Freising, Germany) on a 3100 automated sequencer (Applied Biosystems) using Big Dye-terminators version 3.1 (Perkin Elmer). The cDNA clones for sequencing was derived from mRNA purified from embryos of stages 1 to 13 (based on [26]).

Two 454 libraries were generated by Vertis Biotechnologie AG (Freising, Germany). After Poly(A)+ RNA purification and cDNA synthesis using random hexamer primers, 454 adapters were ligated and the cDNA was amplified by 16 (stages 1–5) or 17 (stages 6–14) PCR cycles. The cDNA was subsequently normalized by one cycle of denaturation and reassociation of the cDNA. Reassociated ds-cDNA was separated from single stranded cDNAs by hydroxypalatite chromatography and finally amplified by 10 (stages 6–14) or 11 (stages 1–5) PCR cycles, before size fractionation by preparative gel electrophoresis to fragments of 500–700bp.

The two samples of total RNA for Illumina sequencing were sent to Macrogen (Seoul, Korea) for library preparation and mRNA sequencing (RNAseq). In summary, enrichment of mRNA with poly-T oligo-attached magnetic beads, fragmentation of purified mRNA, cDNA synthesis and sequencing adapter ligation for library preparation were performed following the standard procedure of the Illumina TruSeq RNA Sample Preparation Kit (Illumina). The two libraries were sequenced separately in two lanes of an Illumina HiSeq2000 sequencing system.

All 454 and Illumina reads generated in this study have been submitted to the NCBI Short Read Archive (BioProject PRJNA253975). And the ESTs generated in this study are available at NCBI (Accession Number JZ713073 - JZ714620).

### De-novo transcriptome assembly

#### Quality assessment and de novo assembly of 454 reads

1,040,006 454 pyrosequencing reads were generated by Vertis Biotechnologie AG (Freising, Germany) (see above). In addition, 22,812 already available ESTs ([20], [21]; FY216297-FY225483 and FY368221-FY381845) were downloaded from NCBI and 1,548 ESTs were newly generated (see above). All 454 reads and ESTs were assembled using Newbler 2.6. The assembly used the (-vs) flag with NCBI UniVec.fasta to remove common contaminant vectors, and the (-vt) flag with adaptors (File S1) to remove residual sequencing adaptors. The assembly also used the (-cdna) flag, which triggers the *de novo* transcriptome assembly algorithm. The (-ml 30) flag was used to limit the minimum alignment overlap to be 30 base pairs.

#### Quality assessment and filtering of Illumina reads

The raw Illumina reads for each library were delivered as individual fastq files for each pair. The two files of the embryonic and post-embryonic sample respectively were interleaved using the shuffleSequences_fastq.pl perl script provided with the *de novo* assembler Velvet (version 1.2.08) [30], resulting in one fastq file for each sample. A custom perl script (IlluminaEndTrim.pl; the script is available on request) was used to trim bases of the 3′ ends that possessed quality scores smaller than 35 (Illumina 1.8+, Phred+33). Similarly, 3′ and 5′ adenine and thymidine runs of more than 10 bases were removed using the perl script IlluminaPolyA.pl (the script is available on request). Since this quality and Poly-A trimming can result in the loss of read pairing due to removal of individual mates, the singletons were removed from the trimmed files (removeSingle.pl, the script is available on request). This procedure resulted in two fastq files for each sample, one with properly matched paired reads and one with single reads. These two files per sample were used for the subsequent *de novo* assemblies.

#### Combined de novo transcriptome assembly

The reads originating from the two Illumina HiSeq2000 lanes and the assembled 454 reads were assembled with Velvet (version 1.2.08) [30] and Oases (version 0.2.08) [31]. Velvet and Oases were compiled with LONGSEQUENCES activated, MAXKMERLENGTH of 53 to allow for assemblies with larger hash lengths and 3 CATEGORIES. Since the full assembly with all data exceeded the computational power of the available 528 GB RAM Server, the two fastq files with paired Illumina reads (see above) were randomly split into three subsets each (randomSplit.pl, the script is available on request). The Illumina data was progressively assembled with the 454 reads in three assemblies (Fig. 1) as follows: For the first assembly, the Illumina reads were flagged in velveth as *–shortPaired* and *–short* for the first third of two interleaved paired fastq files, and the two fastq files with singletons respectively and the already assembled 454 reads were defined as *–long* reads. A value of k = 27 was used as hash length in velveth. In velvetg the insert length was set to 250 bp (*-ins_length 250*) and read tracking was activated as a prerequisite for the subsequent Oases step (*-read_trkg yes*). Furthermore, sequences containing long reads were preserved with the *–conserveLong yes* option, and the merging of polymorphisms was set to less stringent parameters whereby Velvet does not merge two sequences if more than 7 bp of the longer sequences is unaligned (*-max_gap_count 7*). In Oases the insert length was set to 250 bp (*-ins_length 250*) and the reads that were not used for the assembly were preserved by the *-unused_reads yes* option. The output of this first assembly was a fasta file that contains all assembled transcripts and a fasta file with unused reads. Since all 454 reads that were not covered by Illumina reads ended up in the file with unused reads, fragments longer than 110 bp were filtered out for the subsequent assembly using a custom perl script (LengthFilter.pl, the script is available on request).

The second and third assemblies were performed as described above with the second and third of the two interleaved paired fastq files (*-shortPaired*) respectively. The assembled transcripts and the filtered unused reads (longer than 110 bp) from the first and second assembly respectively were defined as long reads (*-long*). Finally, the transcripts that resulted from the last assembly were merged with the assembled reads longer than 110 bp from the unused reads file after final assembly. This assembly (termed Assembly 3, Fig. 1) was the final assembly and was used for subsequent gene prediction and mapping analyses.

### Annotation and assessment of assembly quality

#### Gene prediction, gene ontology and ASGARD database

The gene prediction and gene ontology (GO) annotation was performed as previously described [63]. In summary, all transcriptome sequences were queried against the *D. melanogaster* proteome (FB2011_03 Dmel Release 5.35) using BLASTX and the top 50 hits with an E-value cutoff of 1e-10 were stored in a MySQL database. Similarly, the best TBLASTN hit (E-value cutoff of 1e-10) of the *D. melanogaster* proteome against the *P. tepidariorum* transcriptome were also stored in the same database. This database was used to identify ortholog sequences based on reciprocal top BLAST hits. Paralogous sequences were defined as *P. tepidariorum* transcripts with a top BLAST hit being a *D. melanogaster* gene that does not have it as reciprocal top BLAST hit. Using blast2go v1.2.7 the GO annotations of the top 50 BLAST results with an E-value cutoff of 1e-10 were gathered from the GO database. All annotation data was uploaded to the ASGARD database using previously published scripts [34].

#### Estimation of Sequencing Depth

Progressively larger subsets of reads originating from one Illumina lane (embryonic sample, Sample A) were assembled de novo to estimate the sequencing depth. To this end, the high quality and Poly-A trimmed paired reads and singletons of the embryonic sample were randomly selected to generate seven sub-datasets that were composed of 10% to 70% of all the reads with one paired and one singleton file each (randomSelection.pl, the script is available on request). Those sub-datasets were assembled *de novo* with Velvet (version 1.2.08) [30] and Oases (version 0.2.08) [31] using the following settings for velvetg (*-ins_length 300 -min_pair_count 2 -read_trkg yes -unused_reads yes*) and oases (*-ins_length 300 -min_pair_count 2 -unused_reads yes*), respectively. As described above, the interleaved paired files were defined as *–shortPaired* and the files containing the singleton were defined as *–short*. The number of assembled transcripts in each of the seven sub-assemblies was analyzed by gene prediction (see below).

In addition to the assembly of progressively larger datasets, we estimated the coverage for each transcript in the final comprehensive assembly based on the number of mapped reads. The paired high quality reads (singletons excluded) from the embryonic and postembryonic Illumina libraries were independently mapped against the full assembly (see below). For each transcript in the final assembly the sum of mapped reads from each library was used to calculate the coverage as number of reads divided by the length of the respective transcript. The correlation between transcript length and the number of mapped reads was calculated using R (version 2.15.2) [80]. Note that both a parametric (Pearson correlation) and a non-parametric test (Spearman correlation) were used because it is impossible to test for normal distribution of the data for more than 5,000 samples.

#### Ortholog Hit Ratio analysis and species distribution of best BLAST hits

Ortholog hit ratio (OHR) analysis and species distribution of best BLAST hits was performed as previously described [63]. In summary, all transcripts were queried against the *D. melanogaster* and *I. scapularis* proteome applying BLASTX with an E-value cutoff of 1e-10 and the size of each alignment was scored to calculate the OHR as published [68]. For all transcripts the top BLASTX hit against the non-redundant protein database was used for analysis of the phylogenetic distribution of species of top BLAST hits.

#### Open reading frame, retroelement, protein domain and developmental signaling pathway analyses

Open reading frames for the entire final assembly were predicted using TransDecoder (version: rel16JAN2014) applying default settings (i.e. minimum protein length: 100). Potential retroelements were identified using RepeatMasker (version open-4.0.5) with rmblastn (version 2.2.27+) applying the default mode. The underlying database was RepBase (Update 20140131) and RM database (version 20140131). The protein domain and developmental pathway analyses were performed as previously described [63]. In summary, each assembled *P. tepidariorum* transcript was converted into a predicted protein sequence using ESTSCAN (v-3.0.3). For those protein sequences with a confidence score higher than zero, a protein domain search was performed applying InterPro scan (IPRSCAN, v-4.8). All components of developmental signaling pathways were identified based on the KEGG database and *D. melanogaster,* mouse or human sequences were used to query the annotated *P. tepidariorum* transcriptome using the ASGARD web interface.

### Differential Expression analysis

In order to identify transcripts that are enriched in either of the two Illumina datasets, the reads of the embryonic and the post-embryonic library were independently mapped against the full reference assembly with Bowtie2 (version 2.0.2) [74]. After building a proper index of the reference transcriptome with the *bowtie2-build* function, the high quality and Poly-A trimmed reads were mapped using the preset option *—very-sensitive-local* and allowing for one mismatch (*-N 1*) to increase mapping sensitivity. The resulting alignments were stored in SAM-files (Sequence Alignment/Map file) that were converted into BAM-files (Binary Alignment/Map file) with SAMtools [75]. SAMtools was also used to sort and index the BAM-files and finally to count the number of mapped reads for each transcript in the reference transcriptome (*idxstats* option). Additionally, basic mapping statistics were extracted using the *flagstat*-option of SAMtools.

Since 331,060,788 reads from the embryonic library and only 292,205,142 reads from the post-embryonic library were used for mapping, the raw read count for the embryonic sample was corrected for this difference (corrected read count = raw read count*0.882633, since 292,205,142/331,060,788 = 0.0882633).

All transcripts that had no reads mapped to it in one sample, but at least 1000 read counts in the other sample were defined as over- and underrepresented respectively. Those sequences were extracted from the reference transcriptome and subjected to functional annotation with Blast2GO (version 2.6.2) [76], [77]: The sequences were compared with the non-redundant protein sequence database (nr) using BLASTX and an e-value cutoff of 1e-5. GO terms were associated with BLAST hits that passed this cutoff value. Subsequent GO-term analysis and basic BLAST statistics were also performed with Blast2GO.

## Supporting Information

Figure S1 **Analysis of protein domains.** A All transcripts of the final assembly were compared to nr by BLASTX with an E-value cutoff of 1e-10 (middle). Transcripts with (right) and without (left) BLAST hits were queried against the PROSITE functional protein domain database [46]. B A comparison of identified protein domains of transcripts with (right) and without (left) BLAST hits does not reveal a qualitative difference between both datasets. Protein domains marked in red are specific for each dataset. See also Table S2 for more details.
(TIF)

Figure S2 **Correlation of transcript length and the number of mapped reads.** The Illumina reads were mapped against transcripts of the final assembly and the number of mapped reads was correlated with the length of the respective transcript. This analysis shows a weak positive correlation between transcript length and number of mapped reads.
(TIF)

Figure S3 **Frequency distribution of transcript coverage.** The coverage for each transcript of the final assembly was calculated as number of mapped reads divided by the length of the respective transcript.
(TIF)

Table S1 **Genomic and transcriptomic resources available for chelicerates.**
(PDF)

Table S2 **The protein domains identified in transcripts with (first sub table) and without (second sub table) BLAST hits against the nr database are listed.**
(XLSX)

Table S3 **The components of conserved developmental pathways based on the KEGG database** [57] **are listed.** The number of hits in the spider transcriptome (‘# Hits’), the length of the longest and shortest transcript of BLAST hits (‘Length range’), the query gene, the transcript IDs of the spider transcriptome and the accession number for orthologs that were previously published are given.
(XLSX)

Table S4 **Gene ontology (GO) analysis for embryonically enriched transcripts.**
(XLSX)

Table S5 **Gene ontology (GO) analysis for postembryonically enriched transcripts.**
(XLSX)

File S1 **Adapter sequences for 454 pyrosequencing reads.**
(TXT)
