# Supplementary figures and images for: A Comprehensive Reference Transcriptome Resource for the Common House Spider Parasteatoda tepidariorum
Source: PLoS One. 2014 Aug 13;9(8):e104885. doi: 10.1371/journal.pone.0104885 (PMC4132015; doi:10.1371/journal.pone.0104885)

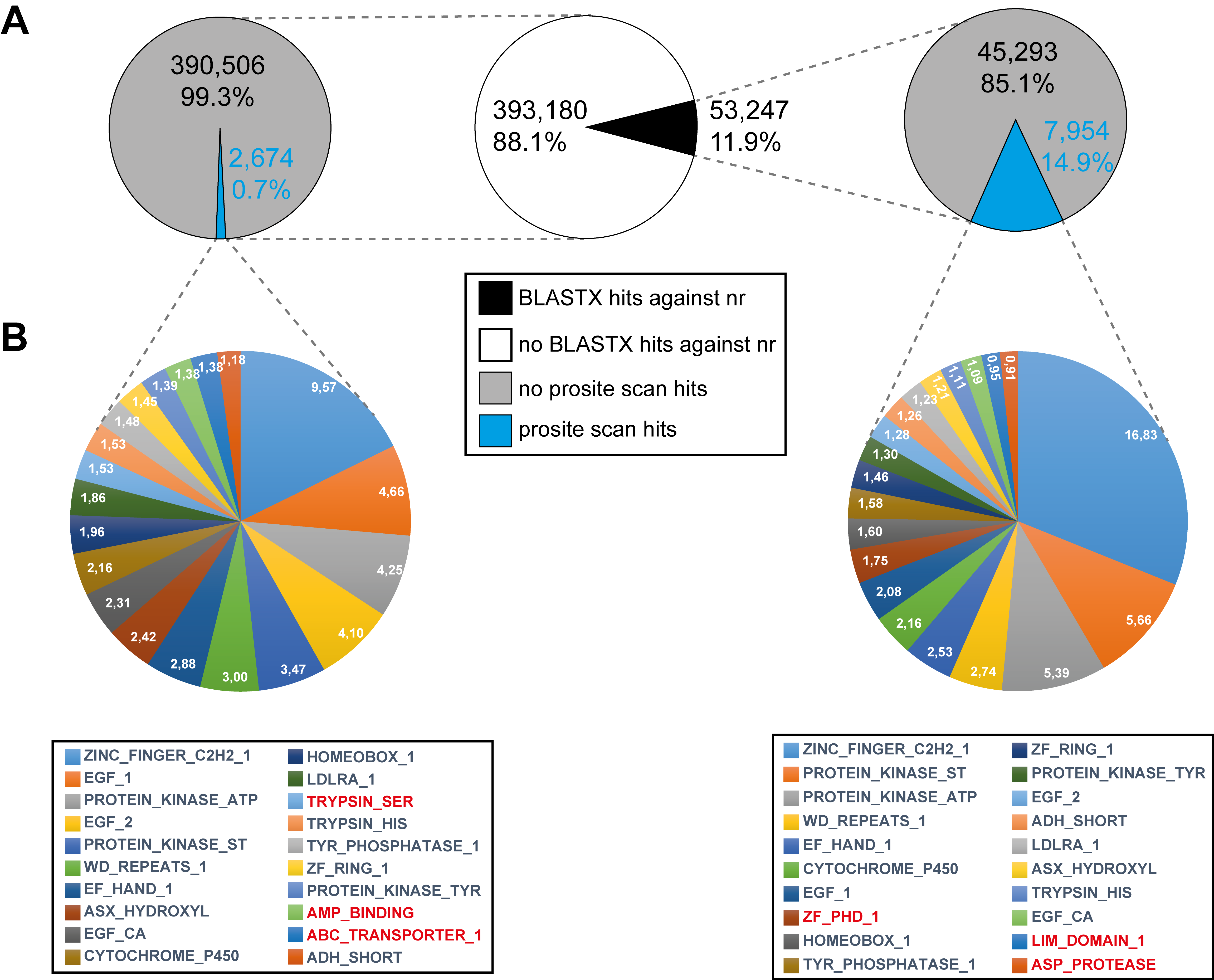

Supplement: Figure S1 — Analysis of protein domains. A All transcripts of the final assembly were compared to nr by BLASTX with an E-value cutoff of 1e-10 (middle). Transcripts with (right) and without (left) BLAST hits were queried against the PROSITE functional protein domain database [46]. B A comparison of identified protein domains of transcripts with (right) and without (left) BLAST hits does not reveal a qualitative difference between both datasets. Protein domains marked in red are specific for each dataset. See also Table S2 for more details. (TIF) [file pone.0104885.s001.tif]

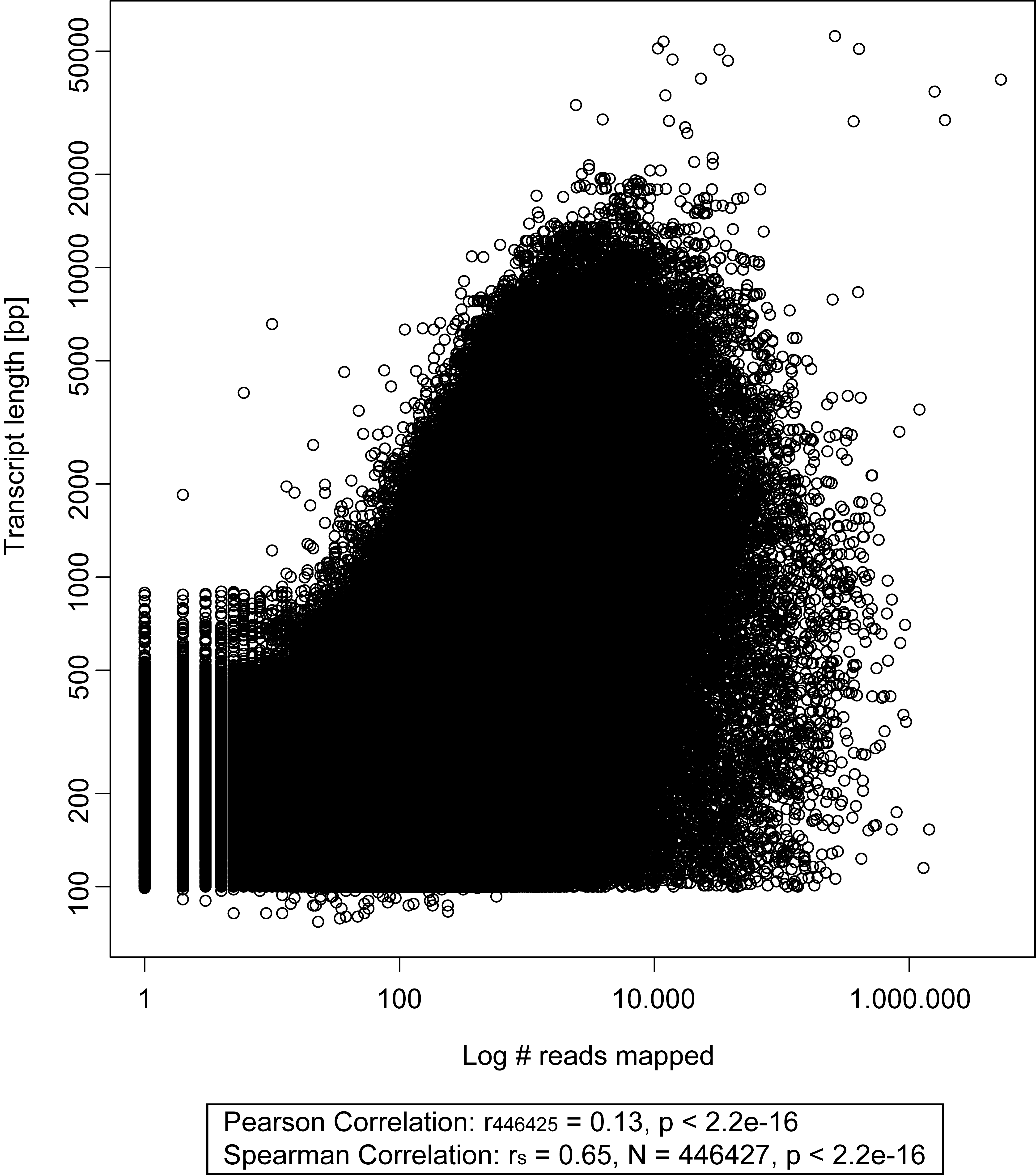

Supplement: Figure S2 — Correlation of transcript length and the number of mapped reads. The Illumina reads were mapped against transcripts of the final assembly and the number of mapped reads was correlated with the length of the respective transcript. This analysis shows a weak positive correlation between transcript length and number of mapped reads. (TIF) [file pone.0104885.s002.tif]

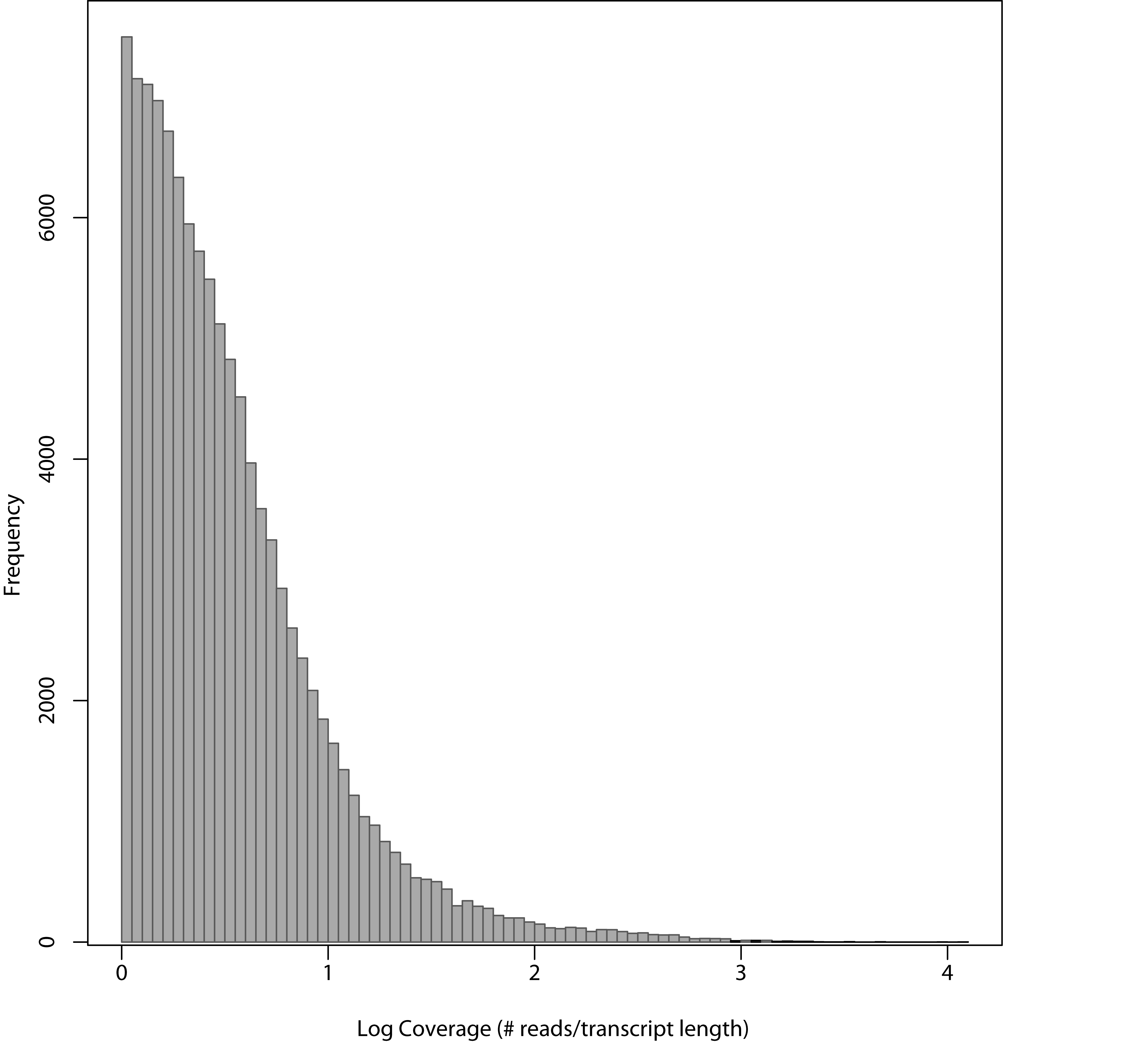

Supplement: Figure S3 — Frequency distribution of transcript coverage. The coverage for each transcript of the final assembly was calculated as number of mapped reads divided by the length of the respective transcript. (TIF) [file pone.0104885.s003.tif]
