# Supplementary material for: A Comprehensive Reference Transcriptome Resource for the Common House Spider Parasteatoda tepidariorum
Source: PLoS One. 2014 Aug 13;9(8):e104885. doi: 10.1371/journal.pone.0104885 (PMC4132015; doi:10.1371/journal.pone.0104885)
Supplement: Table S1 — Genomic and transcriptomic resources available for chelicerates. (PDF) [file pone.0104885.s004.pdf]

Species	Order	Resource	Tissue Origin	# reads	# bp	# contigs/transcripts/ unigenes/gene models	References
Limulus polyphemus	Xiphosura	genome	adult third walking leg, larvae	1,700,000,000	N/A	N/A	[1]
Rhipicephalus (Boophilus) microplus	Acari	BAC library	N/A	3,072	136,474,000	24	[2]
Rhipicephalus (Boophilus) microplus	Acari	EST library	eggs, larvae, nymphs, adults, dissected adult organs	42,512	N/A	13,643	[3,4]
Rhipicephalus appendiculatus	Acari	EST library	salivary gland	18,422	626,000	7,359	[5]
Ixodes scapularis	Acari	genome	N/A	N/A	1,388,472,180	24,925	[6,7]
Tetranychus urticae	Acari	genome	embryos	N/A	~10,000,000,000	18,414	[8]
Mesobuthus martensii	Scorpiones	BAC library	male testis	46,080	46,080,000,000	N/A	[9]
Mesobuthus martensii	Scorpiones	genome	male adult muscle tissues of the pedipalp and metasoma	1,634,795,503	1,128,500,000	32,016	[10]
Loxosceles laeta	Araneae	EST library	venom gland	3,008	N/A	1,357	[11]
Citharischius crawshayi	Araneae	EST library	venom gland	282	N/A	44	[12]
Acanthoscurria gomesiana	Araneae	EST library	hemocytes	7,584	N/A	3,723	[13]
Aphonopelma sp.	Araneae	EST library	leg muscles of adults	2,507	N/A	305	[14]
Chilobrachys jingzhao	Araneae	EST library	venom gland	788	N/A	356	[15]
Ornithoctonus huwena	Araneae	EST library	venom gland	468	N/A	89	[16]
Lycosa singoriensis	Araneae	EST library	venom gland	833	N/A	331	[17]
Dolomedes mizhoanus	Araneae	EST library	venom gland	356	N/A	N/A	[18]
Gasteracantha cancriformis	Araneae	454 Transcriptome	spinning gland of adult females	52,399	14,339,651	25,663	[19]
Actinopus spp.	Araneae	454 Transcriptome	spinning gland of adult females	34,496	8,087,280	18,257	[19]
Stegodyphus lineatus	Araneae	454 Transcriptome	adult	457675	165,220,675	8,944	[20]
Stegodyphus tentoriicola	Araneae	454 Transcriptome	adult	459,994	157,317,948	6,094	[20]
Stegodyphus mimosarum	Araneae	454 Transcriptome	adult	542,510	175,230,730	5,893	[20]
Theridion grallator	Araneae	Illumina Transcriptome	adult females	239,263,493	N/A	104,481	[21]
Theridion californicum	Araneae	Illumina Transcriptome	adult females	250,930,772	N/A	128,391	[21]
Trittame loki	Araneae	454 Transcriptome	female venom glands	N/A	N/A	4,711	[22]
Latrodectus tredecimguttatus	Araneae	Illumina Transcriptome	venom glands	27,605,467	2,484,492,030	34,334	[23]
Latrodectus hesperus	Araneae	Illumina Tag sequencing	female major ampullate silk glands and cephalothorax	25,857,032	N/A	200,603 unique tags	[24]
Latrodectus hesperus	Araneae	Illumina Transcriptome	silk glands, cephalothorax, seven pairs of venom glands	149,000,000	N/A	103,365	[25]

Gasteracantha arcuata	Araneae	Illumina Transcriptome	adults	23,370,495	4,674,099,000	54,871	[26]
Nasoonaria sinensis	Araneae	Illumina Transcriptome	adults	25,346,489	5,069,297,800	101,855	[26]
Gasteracantha hasselti	Araneae	Illumina Transcriptome	adults	16,416,304	3,283,260,800	75,455	[26]
Stegodyphus mimosarum	Araneae	genome	adults	N/A	~2,550,000,000	27,235	[27]
Acanthoscurria geniculata	Araneae	genome	adults	N/A	~6,000,000,000	~70,000	[27]
Parasteatoda tepidariorum	Araneae	EST library	embryos	24,360	16,537,059	N/A	[28,29], this study
Parasteatoda tepidariorum	Araneae	454 Transcriptome	embryos (stages 1-13)	1,040,006	639,294,706	21,959 isotigs	this study
Parasteatoda tepidariorum	Araneae	Illumina Transcriptome	embryos (stages 1-14)	332,245,126	33,556,757,726	N/A	this study
Parasteatoda tepidariorum	Araneae	Illumina Transcriptome	nymphs, larvae, adult female, adult male	292,831,388	29,575,970,188	N/A	this study
Parasteatoda tepidariorum	**Araneae**	**Combined assembly**	**embryos, nymphs, larvae, adults**	**625,219,691**	**61,839,406,064**	**41,766 unique BLAST hits**	this study

References

1. Nossa CW, Havlak P, Yue J-X, Lv J, Vincent KY, et al. (2014) Joint assembly and genetic mapping of the Atlantic horseshoe crab genome reveals ancient whole genome duplication. GigaScience 3: 9. doi:10.1186/2047-217X-3-9.

2. Moolhuijzen PM, Lew-Tabor AE, Morgan JAT, Valle MR, Peterson DG, et al. (2011) The complexity of Rhipicephalus (Boophilus) microplus genome characterised through detailed analysis of two BAC clones. BMC Res Notes 4: 254. doi:10.1186/1756-0500-4-254.

3. Wang M, Guerrero FD, Pertea G, Nene VM (2007) Global comparative analysis of ESTs from the southern cattle tick, Rhipicephalus (Boophilus) microplus. BMC Genomics 8: 368. doi:10.1186/1471-2164-8-368.

4. Guerrero FD, Miller RJ, Rousseau M-E, Sunkara S, Quackenbush J, et al. (2005) BmiGI: a database of cDNAs expressed in Boophilus microplus, the tropical/southern cattle tick. Insect Biochem Mol Biol 35: 585–595. doi:10.1016/j.ibmb.2005.01.020.

5. Nene V, Lee D, Kang'a S, Skilton R, Shah T, et al. (2004) Genes transcribed in the salivary glands of female Rhipicephalus appendiculatus ticks infected with Theileria parva. Insect Biochem Mol Biol 34: 1117–1128. doi:10.1016/j.ibmb.2004.07.002.

6. Hill CA, Wikel SK (2005) The Ixodes scapularis Genome Project: an opportunity for advancing tick research. Trends Parasitol 21: 151–153. doi:10.1016/j.pt.2005.02.004.

7. Vectorbase (n.d.). Available: http://www.vectorbase.org.

8. Grbić M, Leeuwen TV, Clark RM, Rombauts S, Rouzé P, et al. (2011) The genome of Tetranychus urticae reveals herbivorous pest adaptations. Nature 479: 487–492. doi:10.1038/nature10640.

9. Li S, Ma Y, Jang S, Wu Y, Liu H, et al. (2009) A HindIII BAC library construction of Mesobuthus martensii Karsch (Scorpiones:Buthidae): An important genetic resource for comparative genomics and phylogenetic analysis. Genes Genet Syst 84: 417–424. doi:10.1266/ggs.84.417.

10. Cao Z, Yu Y, Wu Y, Hao P, Di Z, et al. (2013) The genome of Mesobuthus martensii reveals a unique adaptation model of arthropods. Nat Commun 4. Available: http://www.nature.com/ncomms/2013/131015/ncomms3602/full/ncomms3602.html. Accessed 12 November 2013.

11. Fernandes-Pedrosa M de F, Junqueira-de-Azevedo I de L, Gonçalves-de-Andrade RM, Kobashi LS, Almeida DD, et al. (2008) Transcriptome analysis of Loxosceles laeta (Araneae, Sicariidae) spider venomous gland using expressed sequence tags. BMC Genomics 9: 279. doi:10.1186/1471-2164-9-279.

12. Diego-García E, Peigneur S, Waelkens E, Debaveye S, Tytgat J (2010) Venom components from Citharischius crawshayi spider (Family Theraphosidae): exploring transcriptome, venomics, and function. Cell Mol Life Sci 67: 2799–2813. doi:10.1007/s00018-010-0359-x.

13. Lorenzini DM, Silva Jr PI da, Soares MB, Arruda P, Setubal J, et al. (2006) Discovery of immune-related genes expressed in hemocytes of the tarantula spider Acanthoscurria gomesiana. Dev Comp Immunol 30: 545–556. doi:10.1016/j.dci.2005.09.001.

14. Zhu J, Sun Y, Zhao F-Q, Yu J, Craig R, et al. (2009) Analysis of tarantula skeletal muscle protein sequences and identification of transcriptional isoforms. BMC Genomics 10: 117. doi:10.1186/1471-2164-10-117.

15. Chen J, Zhao L, Jiang L, Meng E, Zhang Y, et al. (2008) Transcriptome analysis revealed novel possible venom components and cellular processes of the tarantula Chilobrachys jingzhao venom gland. Toxicon Off J Int Soc Toxinology 52: 794–806. doi:10.1016/j.toxicon.2008.08.003.

16. Jiang L, Peng L, Chen J, Zhang Y, Xiong X, et al. (2008) Molecular diversification based on analysis of expressed sequence tags from the venom glands of the Chinese bird spider Ornithoctonus huwena. Toxicon Off J Int Soc Toxinology 51: 1479–1489. doi:10.1016/j.toxicon.2008.03.024.

17. Zhang Y, Chen J, Tang X, Wang F, Jiang L, et al. (2010) Transcriptome analysis of the venom glands of the Chinese wolf spider Lycosa singoriensis. Zool Jena Ger 113: 10–18. doi:10.1016/j.zool.2009.04.001.

18. Jiang L, Liu C, Duan Z, Deng M, Tang X, et al. (2013) Transcriptome analysis of venom glands from a single fishing spider Dolomedes mizhoanus. Toxicon 73: 23–32. doi:10.1016/j.toxicon.2013.07.005.

19. Prosdocimi F, Bittencourt D, da Silva FR, Kirst M, Motta PC, et al. (2011) Spinning Gland Transcriptomics from Two Main Clades of Spiders (Order: Araneae) - Insights on Their Molecular, Anatomical and Behavioral Evolution. PLoS ONE 6: e21634. doi:10.1371/journal.pone.0021634.

20. Mattila TM, Bechsgaard JS, Hansen TT, Schierup MH, Bilde T (2012) Orthologous genes identified by transcriptome sequencing in the spider genus Stegodyphus. BMC Genomics 13: 70. doi:10.1186/1471-2164-13-70.

21. Croucher PJ, Brewer MS, Winchell CJ, Oxford GS, Gillespie RG (2013) De novo characterization of the gene-rich transcriptomes of two color-polymorphic spiders, Theridion grallator and T. californicum (Araneae: Theridiidae), with special reference to pigment genes. BMC Genomics 14: 862. doi:10.1186/1471-2164-14-862.

22. Undheim EAB, Sunagar K, Herzig V, Kely L, Low DHW, et al. (2013) A Proteomics and Transcriptomics Investigation of the Venom from the Barychelid Spider Trittame loki (Brush-Foot Trapdoor). Toxins 5: 2488–2503. doi:10.3390/toxins5122488.

23. He Q, Duan Z, Yu Y, Liu Z, Liu Z, et al. (2013) The Venom Gland Transcriptome of Latrodectus tredecimguttatus Revealed by Deep Sequencing and cDNA Library Analysis. PLoS ONE 8: e81357. doi:10.1371/journal.pone.0081357.

24. Lane AK, Hayashi CY, Whitworth GB, Ayoub NA (2013) Complex gene expression in the dragline silk producing glands of the Western black widow (Latrodectus hesperus). BMC Genomics 14: 846. doi:10.1186/1471-2164-14-846.

25. Clarke TH, Garb JE, Hayashi CY, Haney RA, Lancaster AK, et al. (2014) Multi-tissue transcriptomics of the black widow spider reveals expansions, co-options, and functional processes of the silk gland gene toolkit. BMC Genomics 15: 365. doi:10.1186/1471-2164-15-365.

26. Zhao Y-J, Zeng Y, Chen L, Dong Y, Wang W (2013) Analysis of transcriptomes of three orb-web spider species reveals gene profiles involved in silk and toxin. Insect Sci: n/a–n/a. doi:10.1111/1744-7917.12068.

27. Sanggaard KW, Bechsgaard JS, Fang X, Duan J, Dyrlund TF, et al. (2014) Spider genomes provide insight into composition and evolution of venom and silk. Nat Commun 5. Available: http://www.nature.com/ncomms/2014/140506/ncomms4765/full/ncomms4765.html. Accessed 9 July 2014.

28. Kanayama M, Akiyama-Oda Y, Nishimura O, Tarui H, Agata K, et al. (2011) Travelling and splitting of a wave of hedgehog expression involved in spider-head segmentation. Nat Commun 2: 500. doi:10.1038/ncomms1510.

29. Oda H, Nishimura O, Hirao Y, Tarui H, Agata K, et al. (2007) Progressive activation of Delta-Notch signaling from around the blastopore is required to set up a functional caudal lobe in the spider Achaearanea tepidariorum. Dev Camb Engl 134: 2195–2205. doi:10.1242/dev.004598.
